# ASO Author Reflections: Impact of PIPAC-Oxaliplatin on Functional Recovery, Quality of Life, Good Days, and Survival in Refractory Colorectal and Appendiceal Carcinomatosis

**DOI:** 10.1245/s10434-024-16080-4

**Published:** 2024-12-03

**Authors:** Muhammad Talha Waheed, Nora Ruel, Thanh H. Dellinger, Mustafa Raoof

**Affiliations:** 1 Department of Surgery, City of Hope National Medical Center, Duarte, CA USA; 2 Computation and Quantitative Medicine, City of Hope National Medical Center, Duarte, CA USA

## Past

Peritoneal metastases of colorectal cancer (CRC) or appendiceal cancer (AC) origin are associated with poor survival and cause a significant detriment in health-related quality of life (HRQoL), requiring frequent hospitalizations. While cytoreductive surgery is the mainstay of treatment, most patients do not qualify due to unresectable burden of disease. For these patients, overall survival is estimated to be around 14 months even with the use of modern systemic chemotherapy.^1^ Survival could be as low as 6 months in chemo-refractory settings.^2^ Pressurized intraperitoneal aerosolized chemotherapy (PIPAC) has emerged as an optimized way to deliver intraperitoneal chemotherapy to chemo-refractory patients in an effort to establish locoregional control. Our previous work, the first-in-USA PIPAC-oxaliplatin phase 1 trial,^3^ echoed the safety, feasibility, and efficacy signal demonstrated in prior European studies. Given the novelty of PIPAC, there is a lack of comparative data to determine the benefit of PIPAC over standard-of-care therapy.

## Present

The current study^4^ is the first of its kind to compare the survival and quality of life (using good days) against a matched cohort of patients with peritoneal metastases of CRC or AC origin treated with third-line standard therapy. Good days (days alive and outside hospital) is an objective endpoint used in palliative oncology trials. The present study also looks at HRQoL and functional recovery (using daily step count) for a subset of PIPAC patients. Selection of standard therapy cohort was done meticulously with reporting of all consecutive patients meeting inclusion to reduce selection bias. Patients undergoing PIPAC had significantly lower hospitalizations, higher median number of good days at 6 months (181 versus 131 days) and 1 year (323 versus 131 days), and a higher overall survival (11.3 versus 5.1 months) compared with patients undergoing standard therapy. Although nonsignificant, PIPAC patients also demonstrated a longer progression-free survival (2.9 versus 2.1 months). Consistent with prior studies, patient-centered outcomes (HRQoL) showed no worsening with repeated PIPACs. Step counts declined immediately following PIPAC but returned to baseline within 2–4 weeks.

## Future

The findings from this study provide objective evidence of a promising future for PIPAC. Due to major limitations of a retrospective comparison, these data cannot be used to justify off-label PIPAC as the efficacy remains unproven. The findings are hypothesis generating, and these data provide support for a future randomized trial to objectively evaluate the efficacy of PIPAC. The study also provides the utility of using good days and daily step count data as two possible endpoints in a future randomized trial of PIPAC.
